# CT radiomics from intratumor and peritumor regions for predicting poorly differentiated invasive nonmucinous pulmonary adenocarcinoma

**DOI:** 10.1038/s41598-025-99465-z

**Published:** 2025-04-25

**Authors:** Lijun Duan, Wenyun Liu, Mingyang Li, Liang Guo, Mengran Ren, Xin Dong, Xiaoqian Lu, Dianbo Cao

**Affiliations:** 1 Department of Radiology, The First Hospital of Jilin University, No. 1 Xinmin Street, Changchun, 130021 Jilin China; 2 Department of Pathology, The First Hospital of Jilin University, Changchun, Author’s email, 130021 Jilin China

**Keywords:** Radiomics, Adenocarcinoma of lung, X-ray computed, Radiologists, Diagnostic imaging, Cancer, Cancer imaging, Cancer models, Lung cancer

## Abstract

Per the 2021 World Health Organization (WHO) Classification of Thoracic Tumors, poorly differentiated invasive nonmucinous adenocarcinoma (INMA) demonstrates aggressive clinicopathological behavior characterized by lymphatic invasion, correlates with poor prognosis, and necessitates modifications to surgical planning even in early-stage adenocarcinoma. This study aimed to investigate the predictive value of radiomics features of intratumoral and peritumoral microenvironment in poorly differentiated INMA in the lung. A total of 451 patients with INMA were collected from three hospitals. They were divided into the train cohort (173 grade 1/2; 116 grade 3), internal test cohort (89 grade 1/2; 35 grade 3) and external test cohort (26 grade 1/2; 12 grade 3). The logistic regression analysis was used to establish the clinical and radiomic models. The receiver operating characteristic (ROC) curve and the decision curve analysis (DCA) were used to assess diagnostic performance of different models. The internal test dataset (124 patients) was used to evaluate the radiologists’ performance without and with the assistance of optimal model. Nodule attenuation, consolidation size and consolidation-to-tumor ratio (CTR) meaning the ratio of maximum consolidation to tumor dimensions on axial imaging were independent predictors of poorly differentiated INMA (*p* < 0.05). In the internal test cohort, the area under the curve (AUC) values of the clinical model, the intratumor radiomic model, the combined 3 mm radiomic model, and the combined 5 mm radiomic model were 0.875(95%CI: 0.811–0.938), 0.882(95%CI: 0.807–0.957), 0.907(95%CI: 0.844–0.969) and 0.858(95%CI: 0.783–0.933), respectively. Corresponding results in the external test cohort showed moderate performance across all models: clinical model (AUC: 0.760, 95%CI: 0.603–0.916), intratumor radiomic model (AUC: 0.760, 95%CI: 0.580–0.939), and combined 3 mm/5 mm radiomic model(AUC: 0.772, 95%CI: 0.593–0.952; AUC: 0.766; 95%CIs: 0.580–0.953). Radiologists had higher diagnostic performance and confidence score with the aid of the combined 3 mm radiomic model. The combined 3 mm radiomic model of non-enhanced CT can identify poorly differentiated INMA with excellent performance and improve radiologists to achieve better diagnostic performance.

**Supplementary Information:**

The online version contains supplementary material available at 10.1038/s41598-025-99465-z.

## Introduction

Lung cancer is the leading cause of cancer-related death worldwide up to date, with an estimated 1.8 million deaths worldwide in 2020^1^. Adenocarcinoma is the most common histopathological type of non-small cell lung cancer^2^. In May 2021, the 5th edition of the WHO classification of thoracic tumor was released^3^, in which a novel histopathological grading system for INMA was proposed by the International Association for the Study of Lung Cancer(IASLC) in 2020^4^. The new system is graded according to predominant plus high-grade histologic pattern with a cutoff of 20% for the latter^4^, and the high-grade histologic patterns include solid, micropapillary, or complex glandular types, which are further classified as poorly differentiated INMA. The poorly differentiated grade was associated with poor prognosis and elevated risk of lymph node metastasis, whereas the well- and moderately-differentiated grade correlated with improved outcomes^5,6^ and demonstrated significant associations with disease-free survival^7,8^. Furthermore, studies have demonstrated that differentiation grade impacts surgical approach selection^9,10^. Patients with poorly differentiated lung adenocarcinoma are recommended for lobectomy, whereas those with early-stage well- or moderately-differentiated carcinomas may undergo sublobar resection following comprehensive pathologic evaluation of lymph nodes. While current guidelines provide no definitive recommendations regarding postoperative adjuvant chemotherapy administration for lung adenocarcinoma patients across differentiation grades, emerging evidence suggests that those with poorly differentiated grade may derive survival benefits from mentioned treatment^10,11^. Therefore, early identification of poorly differentiated INMA enables prognostic evaluation and guides clinical management.

Currently, as the diagnosis of an invasive component and its histologic pattern cannot be definitively reached in the context lacking the entire tumor sampling, noninvasive evaluation for the histologic patterns primarily relies on preoperative CT imaging^3^. Previous studies indicated tumor size, solid tumor size, consolidation to tumor ratio (CTR) on CT imaging associated with invasive lung adenocarcinoma with micropapillary or solid components^12^. Chen et al.^13^ had found that intratumoral radiomic textures could effectively predict high-grade (micropapillary and solid) components in lung adenocarcinoma. According to revised classification^3^, there were also studies^14,15^ involving using low-dose CT radiomic method to achieve the pathological grading of INMA. Tumor microenvironment(TME) probably means the peritumoral regions consisting of parenchyma immediately surrounding the tumors, and its role has been increasingly recognized in defining biologic tumor behavior including aggressiveness, metastatic potential, and therapy response in the field of cancer biology and oncology^16^. Beig et al.^17^ expanded the application of the tumor microenvironment concept to include the distinction of malignant versus benign lung nodules and demonstrated that the peritumoral 5 mm radiomic model is the most effective in differentiating benign from malignant nodules. Studies^18,19^have demonstrated that the peritumoral 3/5 mm region exhibits advantages in predicting prognosis and EGFR gene mutations in non-small cell lung cancer. Consequently, we conducted an in-depth exploration of the peritumoral shape and textural patterns of heterogeneity within the 3 mm and 5 mm peritumoral ranges, and our study aims to investigate the predictive value of radiomic features based on non-enhanced CT from intratumoral and peritumoral 3 mm/5 mm microenvironment for poorly differentiated INMA.

## Methods

### Patients

**Fig. 1:**
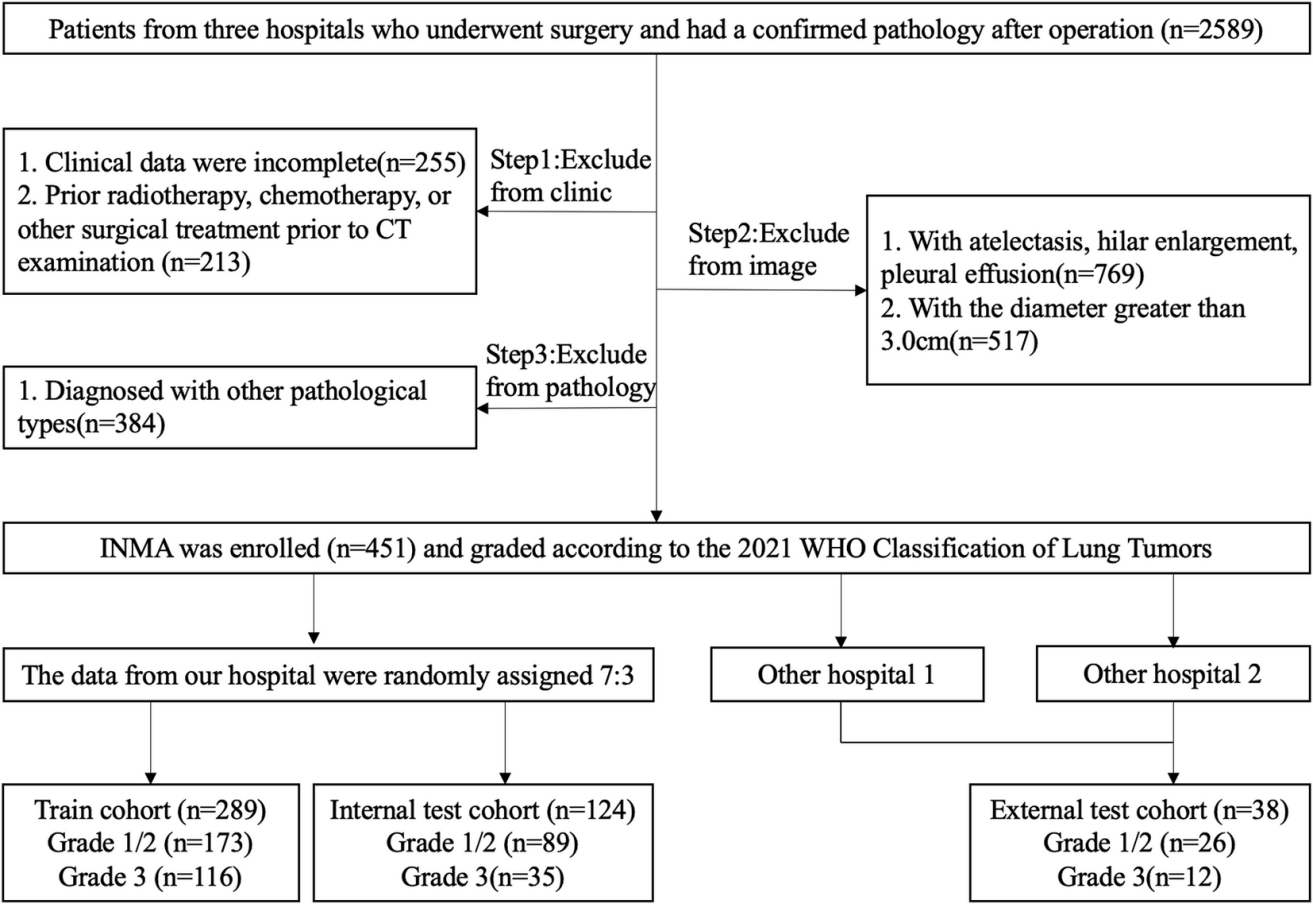
Data inclusion flowchart. Three hospitals include the First Hospital of Jilin University, the Liaoyuan Central Hospital and the Meihekou Central Hospital.

The total of 451 patients (male: female = 177: 274; average age, 59.22 ± 9.00 years; range 29–82 years) with 451 nodules, who had routine CT scan within two weeks before surgery and were histopathologically confirmed to be pulmonary INMA, were retrospectively enrolled into this study from January 2019 to July 2022. The inclusion criteria were: (1) The clinical imaging and pathological data of the patients were complete; (2) The pathological result was defined to be invasive non-mucinous adenocarcinoma after surgery, and the histological sub-type information of IASLC/ATS/ERS was available; (3) Chest CT showed that the axially maximal diameter of the lesion was ≤ 3.0 cm. The exclusion criteria were: (1) the pathological types were other benign and malignant lesions such as granulomatous nodule, invasive mucinous adenocarcinoma, et al.; (2) Patients with atelectasis, hilar enlargement, pleural effusion; (3) Patients with a history of receiving radiotherapy, chemotherapy or other surgical treatment before CT examination. The patients who met these standards of the enrollment from three hospitals were shown in Fig. 1.

### Histopathological evaluation

All pathologic sections were reviewed according to the proposed IASLC grading scheme^20,21^ by a senior pathologist (L.G., with 15 years of experience). The percentage of each histologic pattern should be recorded in 5–10% increments to determine the predominant histologic pattern (sub-type) and quantify any patterns to determine the tumor grade. The grading criteria are listed as following: (1) Grade 1 (well differentiated): focal lesion (≤ 3.0 cm) is composed of lepidic predominant with no or <20% high-grade pattern; (2) Grade 2 (moderately differentiated): focal lesion (≤ 3.0 cm) is made up of acinar or papillary predominant with no or ≥20% high-grade pattern; (3) Grade 3 (poorly differentiated): focal lesion (≤ 3.0 cm) consists of any tumor with≥20% high-grade pattern. High-grade patterns include micropapillary, solid, cribriform, and complex glandular patterns. Meanwhile, the pathological information including lymph node metastases, pleural infiltrates, vascular infiltrates, bronchial infiltrates will be evaluated.

### Image acquisition and analysis

CT imaging of all subjects was performed on PHILIPS iCT64/256, SIEMENS Cardiac 64 and GE Revolution 64 spiral CT machines, ranging from the apical level of the lung to the level of bilateral suprarenal glands. The parameters of the non-contrast-enhanced CT scan were as follows: tube voltage, 110-120kvp; tube current automatically adjusted; pitch, 5; matrix, 521 × 521; reconstruction thickness, 1.0–1.5 mm and viewed on standard lung window (width 1500 HU; level − 600 HU) and mediastinal window (width, 400 HU; level 30 HU) for image observation. After scanning, all CT images were transmitted and stored in the Picture Archiving and Communication System (PACS) with digital imaging and communications in medicine (DICOM) format.

All patients’ CT data were reviewed on a PACS workstation and analyzed by two senior chest radiologists (L.D. and X.L., with 6 years and 11 years of experience) who were blinded to the pathological results. When the conclusions were inconsistent, agreements were reached by consulting a third radiologist with 28 years of experience (D.C.). Observations of interest included: (a) lobar position; (b) nodule attenuation: solid or subsolid nodule; (c) tumor or consolidation size: the maximum diameter of the axial section of a nodule was measured on the standard lung or mediastinal window, respectively; (d) CTR: ratio of maximal consolidation size to maximal tumor size at axial position; (e) nodule features: according to the Fleischer’s guidelines^22^ to determine whether there are spiculation, lobulation, vacuolation, vascular shadow, coursing relationship between bronchus and nodule, pleural indentation.

### Image segmentation and radiomic feature extraction

**Fig. 2:**
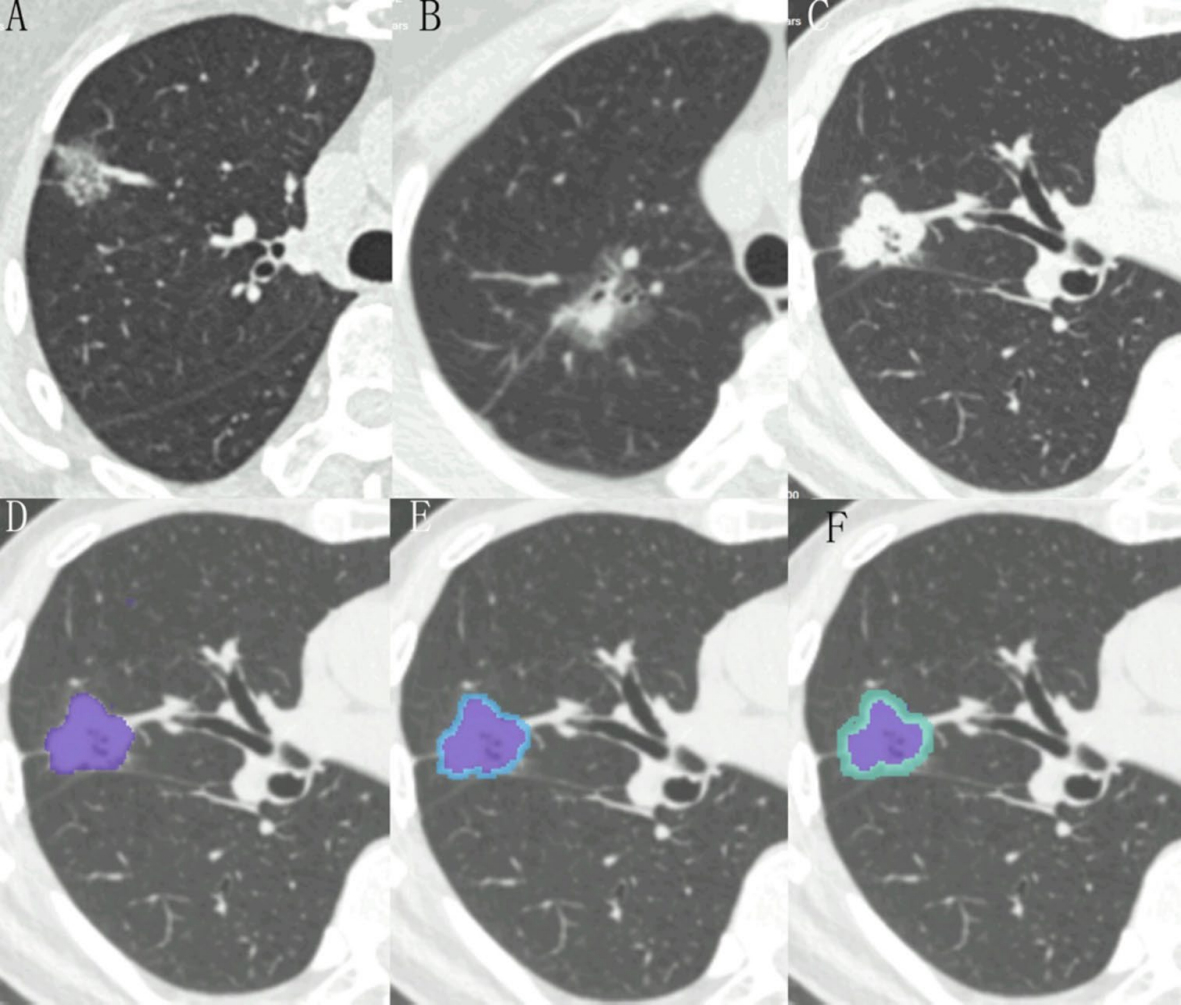
(**A**) A 71-year-old female with grade 1 (lepidic 70%+acinar 30%) has a subsolid nodule, whose CTR is 0.29 and consolidation size is 0.70 cm. P clinic is 0.095. P combined 3 mm is 0.251. (**B**) A 54-year-old female with grade 2 (acinar 70% + lepidic 30%) has a subsolid nodule, whose CTR is 0.50 and consolidation size is 1.50 cm. P clinic is 0.124, P combined 3 mm is 0.054. (**C**) A 67-year-old male with grade 3 (solid 50% + papillary 40% + acinar 10%) has a solid nodule, whose CTR is 0.90 and consolidation size is 2.70 cm. P clinic is 0.875. P combined 3 mm is 0.939. (**D**–**F**) They refer to the schematic diagram of the extent of the intratumor, the combined 3 mm (blue indicates 3 mm peritumoral region) and the combined 5 mm (green indicates 5 mm peritumoral region) ROI using nodule C as a sample.

The regions of interest (ROIs) were manually segmented layer by layer using the RIASEG (https://github.com/lisherlock/RIASEG)^23^ on standard lung window by two radiologists(L.D. and X.D., with 6 years and 5 years of experience), who were blind to the pathological results. After that the masks and DICOM images of the intratumor were introduced into the RIASEG to expand the peritumoral 3 mm and 5 mm respectively, and then we manually erased the areas outside the lung tissue layer by layer (such as chest wall soft tissue, ribs, mediastinal soft tissue, blood vessels, main bronchus, etc.). Finally, the intratumor and the combined 3–5 mm masks were saved in their own folders in the format of “.nii” (Fig. 2) (The combined 3–5 mm means the intratumor and peritumoral 3–5 mm lung tissue regions). After two radiologists independently completed the labeling of all lesions, a repeat delineation was performed on randomly selected 50 lesions one month later to calculate intraclass correlation coefficients(ICC).

Radiomic feature standardization and extraction were performed using the RIAS software (https://github.com/lisherlock/RIAS or https://github.com/lisherlock/RMIT)^24^. B-spline interpolation implemented in RIAS achieved isotropic resampling (1 × 1 × 1 mm³ voxel spacing), ensuring spatial consistency across heterogeneous imaging protocols. This investigation employed wavelet transform and Laplacian of Gaussian (LoG) filtering to extract higher-order texture features. Feature selection was performed through a two-stage process to ensure robustness and biological relevance. First, features demonstrating excellent inter-observer reproducibility (ICC > 0.75) across multiple delineations were retained. Subsequently, we implemented the least absolute shrinkage and selection operator (LASSO) regression^25^, employing ten-fold cross-validation to optimize the λ parameter.

### Radiomic model

The data from our hospital was divided in two datasets: cross-validation and internal test datasets, with a 7:3 proportion. The logical regression (LR) radiomic model using these selected radiomic features was established in RIAS platform, and the five-fold cross-validation method was used to reduce overfitting and parameter tuning in the cross-validation cohort .The performance of the built radio mic models was evaluated with discrimination and clinical application in both internal and external testing cohorts. By comparing these parameters, the optimal radiomic model was screened from the intratumor, combined 3–5 mm radiomic models.

### Subjective evaluation

The pathological grade of 124 patients’ INMA in the internal test cohort were re-evaluated by three radiologists. Two junior radiologists (M.R. and X.D., with 4 years and 5 years of experience) and one senior radiologist (X.L., with 11 years of experience) who were blinded to the pathological results made diagnosis (the 1/2 grade or 3 grade INMA) by reading chest CT images and recorded their self-confidence in establishing final diagnosis. The individual confidence in the diagnosis was scored using the 5-point Likert scale (1 = very certain diagnosis, 2 = certain diagnosis, 3 = likely diagnosis, 4 = uncertain diagnosis, 5 = very uncertain “ambiguous” diagnosis). Subjective evaluation was then repeated by incorporating the result of the optimal radiomic model. The overall accuracy, sensitivity, and specificity of the diagnosis were assessed.

### Statistical analysis

Demographic characteristics were compared between 1/2 grade and 3 grade INMA groups by using the χ^2^ test or Fisher’s exact test for categorical variables and the t-test for continuous variable. The stepwise multivariable ordinal logistic regression analysis was used to elect meaningful clinical and imaging features to establish the clinical model.

The ROC curves were plotted to assess the diagnostic performance of the clinical model and different radiomic models in discriminating 3 grade INMA from 1/2 grade in each cohort. The optimal cutoff of the biomarkers calculated from the training cohort was applied in the testing cohorts. The SHAP was plotted to intuitively explain the results of the model’s predictions. DeLong testing was used to compare the AUC values between internal and external testing cohorts. The DCA curves was used to assess the clinical usefulness of the built models by quantifying the net benefits at different threshold probabilities in the testing cohorts. Fleiss’ kappa consistency test (0 ~ 0.4 fair, 0.4 ~ 0.6 moderate, 0.6 ~ 0.8 substantial, 0.8 ~ 1.0 excellent) is used to evaluate the intra- and inter- reader’s reliability.

The statistical analysis was performed through MedCalc (version 18.2.1), and SPSS software (version 26.0). A 2-sided tailed of *p* < 0.05 was considered statistically significant.

### Ethical statement

We confirm that all methods were carried out in accordance with relevant guidelines and regulations. This study is a retrospective analysis and has been approved by the ethical committee of the First Hospital of Jilin University (No. AF-IRB-032-06). We confirming that informed consent was obtained from all subjects and/or their legal guardian(s).

## Results

### Patient characteristics

A total of 413 pulmonary nodules pathologically confirmed INMA (male : female = 159:254; median age 61.00 (54.00–66.00) years; range 29–82 years) were included in the present study and were further assigned to either the train cohort or test cohort. Of the 413 pulmonary nodules, 70% (*n* = 289) assigned to the train cohort by random sampling: 262 were grade1/2 and 151 were grade 3. There was no statistically significant difference in the clinical characteristics between the grade 1/2 and grade 3, as shown in Table 1. In addition, 38 INMA patients (male: female = 18: 20; average age 60.82 ± 8.59 years; range 41–75 years) were included from other hospitals as the external test cohort according to the same criteria, 26 with grade 1/2, 12 with grade 3, as shown in Table S1.

**Table 1:** Summary of characteristics in train and internal test cohorts.

Characteristics	Train cohort (*n* = 289)			Internal test cohort(*n* = 124)			P^*^value
	Grade 1/2	Grade 3	*P* Value	Grade 1/2	Grade 3	*P* Value	
	(*n* = 173)	(*n* = 116)		(*n* = 89)	(*n* = 35)		
Gender							
F	115 (66.5)	57 (49.1)	0.003*	63 (70.8)	19 (54.3)	0.081	
M	58 (33.5)	59 (50.9)		26 (29.2)	16 (45.7)		0.115
Age (years)	61.00 (54.00–66.00)	61.00 (54.25-67.00)	0.538	60.00 (52.50–65.50)	60.00 (53.00–69.00)	0.590	0.569
With smoking history	48 (27.7)	34 (29.3)	0.772	25 (28.1)	8 (22.9)	0.553	0.806
Lobar position							0.583
Right upper lobe	67 (38.7)	36 (31.1)	0.181	29 (32.6)	11 (31.4)	0.901	
Right middle lobe	9 (5.2)	5 (4.3)	0.729	5 (5.6)	3 (8.6)	0.844	
Right lower lobe	21 (12.2)	23 (19.8)	0.075	13 (14.6)	8 (22.9)	0.270	
Left upper lobe	53 (30.6)	26 (22.4)	0.124	33 (37.1)	5 (14.2)	0.013*	
Left lower lobe	23 (13.3)	26 (22.4)	0.043*	9 (10.1)	8 (22.9)	0.063	
Nodule attenuation							0.223
Solid nodule	35 (20.2)	102 (87.9)	< 0.001*	20 (22.5)	26 (74.3)	< 0.001*	
Subsolid nodule	138 (79.8)	14 (12.1)		69 (77.5)	9 (25.7)		
Tumor size (cm)	2.00 (1.50–2.50)	2.20 (1.80–2.70)	0.005*	1.80 (1.50–2.20)	2.50 (2.00-2.90)	< 0.001*	0.264
Consolidation size (cm)	0.70 (0.00-1.50)	1.90 (1.50–2.40)	< 0.001*	0.60 (0.00-1.20)	2.00 (1.40–2.50)	< 0.001*	0.055
CTR (consolidation tumor ratio)	0.33 (0.00-0.77)	0.90 (0.75-1.00)	< 0.001*	0.27 (0.00-0.71)	0.90 (0.72–0.96)	< 0.001*	0.115
Lobulation	119 (68.8)	104 (89.7)	< 0.001*	58 (65.2)	31 (88.6)	0.009*	0.335
Spiculation	68 (39.3)	55 (47.4)	0.172	26 (29.2)	16 (45.7)	0.081	0.123
Vacuolation	46 (26.6)	27 (23.3)	0.525	29 (32.6)	12 (34.3)	0.856	0.153
Pleural indentation	80 (46.2)	67 (57.8)	0.055	39 (43.8)	15 (42.9)	0.922	0.209
Coursing relationship between bronchus and nodule							
Normal	33 (19.1)	5 (4.3)	< 0.001*	12 (13.5)	2 (5.7)	0.360	0.719
Abnormal	140 (80.9)	111 (95.7)		77 (86.5)	33 (94.3)		
Vascular shadow	120 (69.4)	100 (86.2)	0.001*	53 (59.6)	29 (82.9)	0.014*	0.057
Pathological information							
Lymph node metastases	3 (1.7)	28 (24.1)	< 0.001*	0 (0.0)	10 (28.6)	< 0.001*	0.516
Pleural infiltrates	49 (28.3)	51 (44.0)	0.006*	22 (24.7)	14 (40.0)	0.092	0.322
Vascular infiltrates	3 (1.7)	27 (23.3)	< 0.001*	0 (0.0)	10 (28.6)	< 0.001*	0.584
Bronchial infiltrates	29 (16.8)	39 (33.6)	0.001*	18 (20.2)	9 (25.7)	0.505	0.794

*P < 0.05 means that the difference between the two cohorts was statistically significant.

### Radiomic feature selection and model construction

**Fig. 3:**
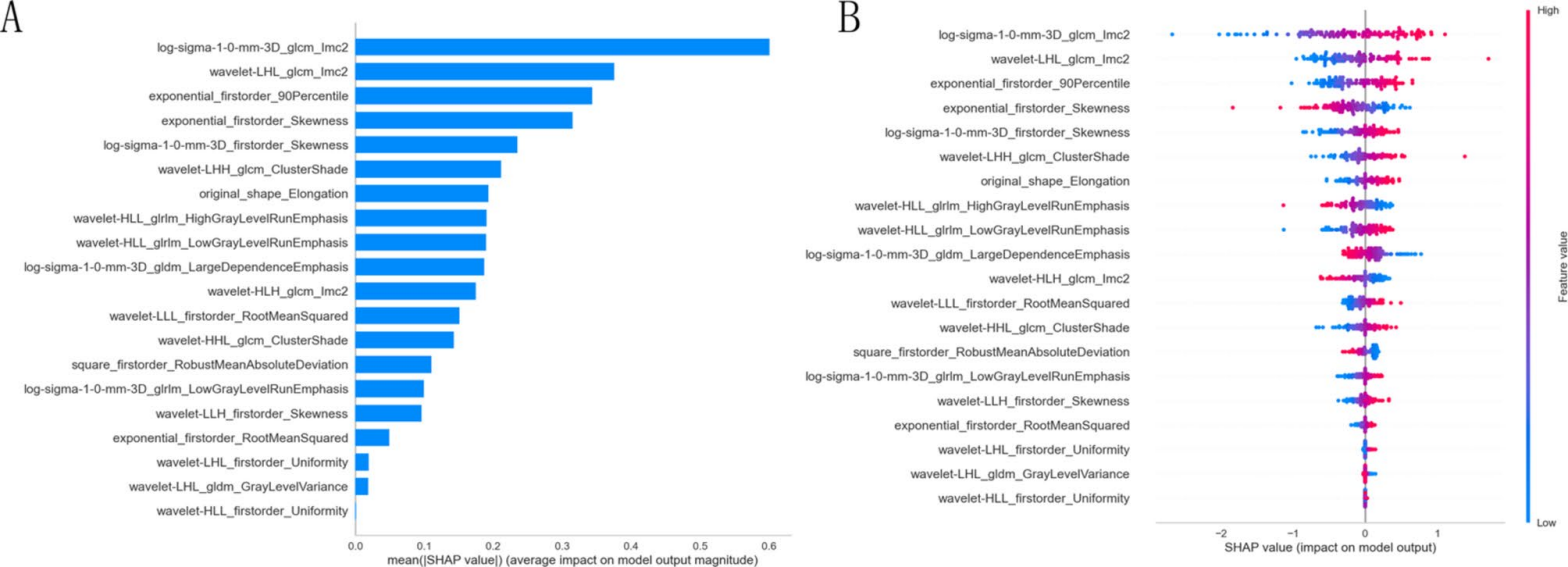
In the internal test cohort, SHAP plots (**A**) shows the 21 features (only the top 20 features are shown in importance) are sorted according to the importance of the output of the combined 3 mm radiomic model and displays the mean SHAP value of every feature. Notably, multi-scale features, texture features, and first-order statistical features emerged as the most critical contributors to model establishment, as identified through our feature importance analysis. The higher the SHAP value, the greater the influence on the output of the model. The SHAP summary plots (**B**) combine feature importance with its influence and show the directionality of each feature.

A total of 999, 1400 and 1020 features, including shape-based features, first-order histogram features, high-order textural features, and transformed features, were obtained based on the ROIs from the intratumor, the combined 3 mm and the combined 5 mm region, separately. After feature reduction, 8 features, 21 features and 16 features were retained to construct the intratumor radiomic model, the combined 3–5 mm radiomic model, respectively. Figure 3 showed the importance of each feature parameter with regard to the prediction of the combined 3 mm radiomic model. The SHAP plot assigns a SHAP value to each feature to quantify its contribution to predictions. A positive SHAP value associated with higher levels of a feature (e.g., log-sigma-1-0-mm-3D_glcm_lmc2) indicates that this feature increases the model’s likelihood of predicting poorly differentiated INMA. Conversely, a negative SHAP value linked to lower levels of a feature (e.g., exponential_firstorder_RootMeanSquared) suggests it reduces the model’s confidence in predicting poorly differentiated INMA.

### Clinical feature selection and model construction

After stepwise multivariable ordinal logistic regression analysis, nodule attenuation, consolidation size, and CTR, which were independent factors for predicting poorly differentiated INMA in the train cohort (*p* < 0.05), were selected to build the clinical model (Table S2).

The clinical model is: T_c_ = ex/(1 + ex), X= -2.234 + 3.676 × nodule attenuation-2.614 × CTR + 1.057 × consolidation size.

The solid nodule is 1 and the subsolid is 0. The cutoff value is 0.589. The *p* > 0.589 is considered to be grade 3 INMA, while *p* < 0.589 is considered to be 1/2 INMA.

### Diagnostic performance of models and radiologists

**Fig. 4:**
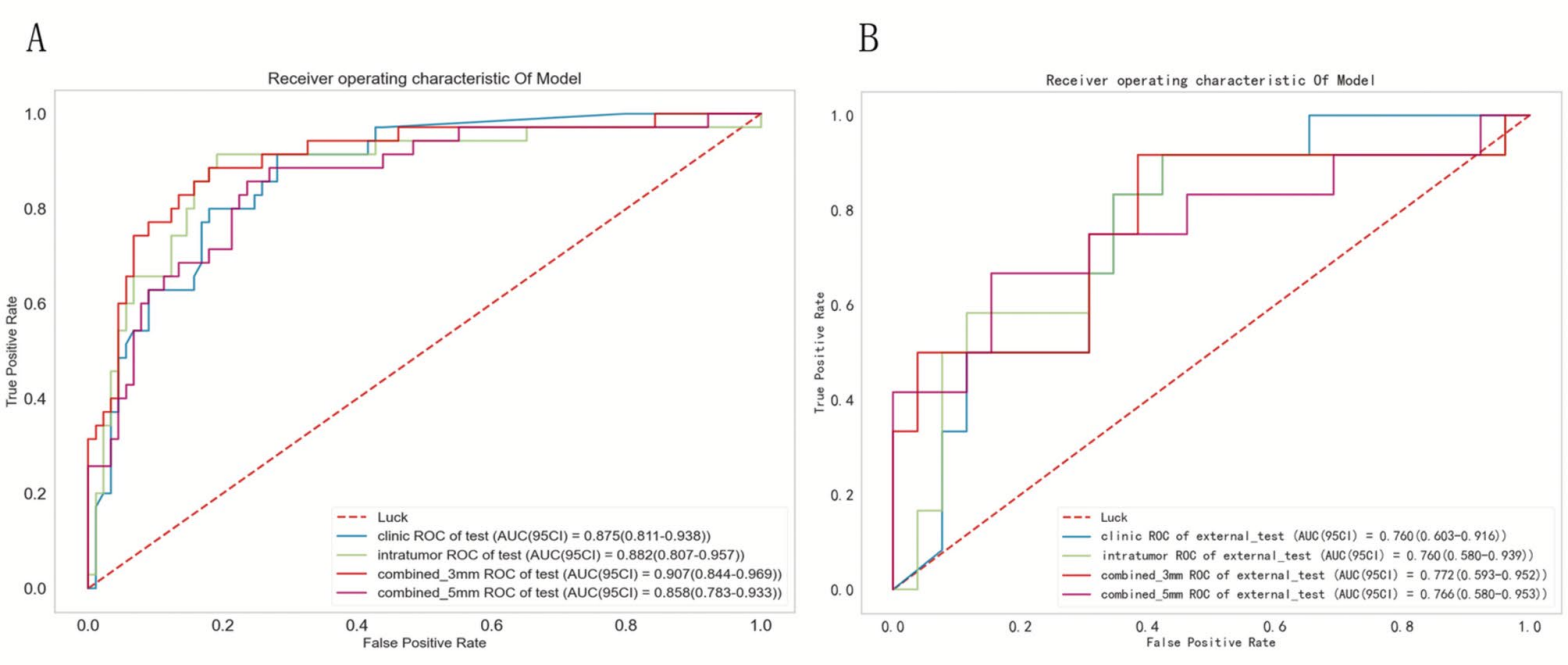
ROC curves for clinic, intratumor, combined 3 mm and combined 5 mm in the internal test cohort (**A**) and external test cohort (**B**).

In the internal test cohort, the AUC values of the clinical model, the intratumor radiomic model, and the combined 3–5 mm radiomic model were 0.875(95%CI 0.811–0.938), 0.882(95%CI 0.807–0.957), 0.907(95%CI 0.844–0.969), and 0.858(95%CI 0.783–0.933), the accuracy was 0.766(95%CI 0.682–0.837), 0.839(95%CI 0.759–0.896), 0.855(95%CI 0.778–0.909), 0.790(95%CI 0.706–0.856), respectively. In the external test cohort, the AUC of the four models were 0.760(95%CI 0.603–0.916), 0.760(95%CI 0.580–0.939), 0.772(95%CI 0.593–0.952), 0.766(95%CI 0.593–0.952), the accuracy was 0.737(95%CI 0.569–0.866), 0.789(95%CI 0.622–0.899), 0.816(95%CI 0.651–0.917), 0.763(95%CI 0.594–0.880), respectively (Table 2; Fig. 4).

**Table 2:** Diagnostic performance of radiomic model, clinical model, and three radiologists for predicting the differentiation grade of INMA.

	AUC (95%CI)	Accuracy (95%CI)	Specificity (95%CI)	Sensitivity (95%CI)
Internal test cohort				
The clinical model	0.875 (0.811–0.938)	0.766 (0.682–0.837)	0.753 (0.650–0.838)	0.800 (0.631–0.916)
The intratumor radiomic model	0.882 (0.807–0.957)	0.839 (0.759–0.896)	0.876 (0.786–0.934)	0.743 (0.564–0.868)
The combined_3mm radiomic model	0.907 (0.844–0.969)	0.855 (0.778–0.909)	0.876 (0.786–0.934)	0.800 (0.625–0.909)
The combined_5mm radiomic model	0.858 (0.783–0.933)	0.790 (0.706–0.856)	0.831 (0.734-0.900)	0.686 (0.506–0.826)
Without AI assistance				
Junior radiologist 1	0.666 (0.558–0.773)	0.669 (0.579–0.751)	0.674 (0.567–0.770)	0.657 (0.478–0.809)
Junior radiologist 2	0.694 (0.588-0.800)	0.710 (0.621–0.788)	0.730 (0.626–0.819)	0.657 (0.478–0.809)
Senior radiologist 3	0.765 (0.671–0.859)	0.750 (0.664–0.823)	0.730 (0.626–0.819)	0.800 (0.631–0.916)
With AI assistance				
Junior radiologist 1	0.821 (0.733–0.910)*	0.831 (0.753–0.892)	0.843 (0.750–0.911)	0.800 (0.631–0.916)
Junior radiologist 2	0.827 (0.739–0.915)*	0.839 (0.762–0.899)	0.854 (0.763–0.920)	0.800 (0.631–0.916)
Senior radiologist 3	0.850 (0.770–0.930)*	0.847 (0.771–0.905)	0.843 (0.750–0.911)	0.857 (0.697–0.952)
External test cohort				
The clinical model	0.760 (0.603–0.916)	0.737 (0.569–0.866)	0.692 (0.482–0.857)	0.833 (0.516–0.979)
The intratumor radiomic model	0.760 (0.580–0.939)	0.789 (0.622–0.899)	0.923 (0.734–0.987)	0.500 (0.223–0.777)
The combined_3mm radiomic model	0.772 (0.593–0.952)	0.816 (0.651–0.917)	0.961 (0.784–0.998)	0.500 (0.223–0.777)
The combined_5mm radiomic model	0.766 (0.593–0.952)	0.763 (0.594–0.880)	0.885 (0.687–0.970)	0.500 (0.223–0.777)

*Referring to *P* < 0.05. Radiologists of all seniorities were more advanced in diagnosis with the assistance of the AI model.

The DeLong test revealed that the combined 3 mm radiomic model demonstrated significantly superior diagnostic efficacy compared to the combined 5 mm radiomic model (AUC: 0.907 vs. 0.858, *P* = 0.005). However, when evaluated against the clinical model (ΔAUC = 0.032, *P* = 0.330) and the intratumor radiomic model (ΔAUC = 0.025, *P* = 0.166), the observed differences failed to reach statistical significance. Within the internal test cohort, the 3 mm combined model demonstrated exceptional diagnostic accuracy (0.855) and specificity (0.876), achieving the highest AUC value (0.907) among all comparative models. In external test cohort, while maintaining superior AUC value (0.772), accuracy(0.816) and specificity (0.961), its sensitivity was lower than that of the clinical model (0.500 vs. 0.833), potentially attributable to the limited external data volume and heterogeneities in acquisition parameters. Based on this evidence, we conclude that the combined 3 mm radiomic model provides better diagnostic efficiency. The typical cases were shown in Fig. 2A-C.

**Fig. 5:**
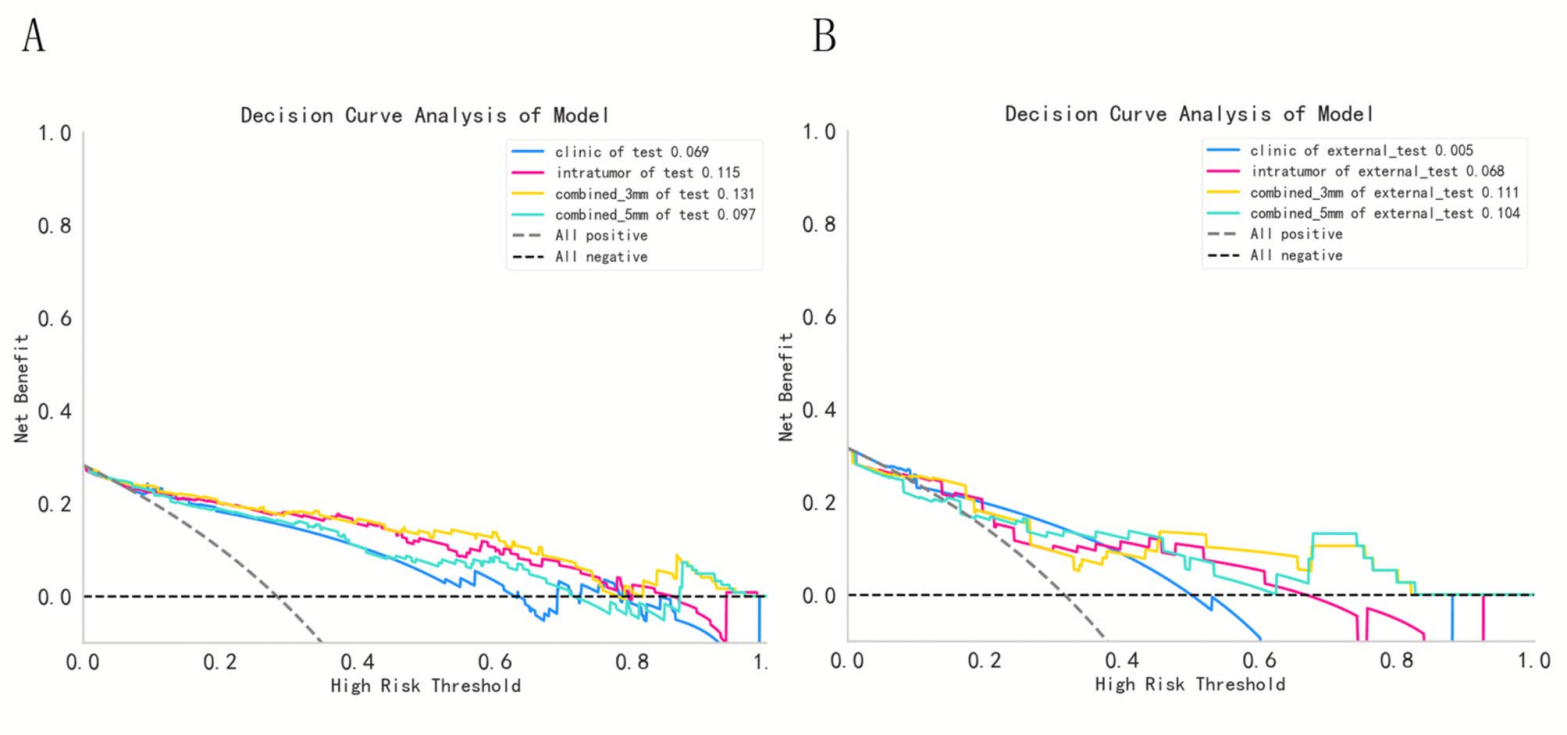
DCA for clinic, intratumor, combined 3 mm and combined 5 mm in the internal test cohort (**A**) and external test cohort (**B**).

DCA was presented in Fig. 5. DCA showed that using the combined 3 mm radiomic model increased more benefit than the treat all project or the treat none project if the threshold probability of a patient or doctor was ˃ 10%, and it had a higher net benefit compared to the other methods. As well as little overlaps within a range from 0.1 to 1.0, the curve of combined 3 mm was always at the top right.

Three radiologists read 124 cases’ CT images in the internal test cohort to evaluate the differentiation degree of lesions with or without the combined 3 mm radiomic model. In the absence of artificial intelligence (AI) model (AI model refers to the combined 3 mm radiomic model), the diagnostic efficiency of senior radiologist 3 was higher than that of junior radiologist 1 (*p* = 0.045), but there was no significant difference between junior radiologist 2 (*p* = 0.204), as shown in Table 2. With the assistance of AI model, the diagnostic efficiency of the three radiologists was improved (radiologist 1: *p* < 0.001; radiologist 2: *p* = 0.005 ; radiologist 3: *p* = 0.001), there was no significant difference in diagnostic efficiency between junior and senior radiologists. The diagnostic accuracy, sensitivity and specificity were all improved, and the average confidence of diagnosis was increased (Table S3). Without the aid of AI model, the consistency of evaluation among the three radiologists was moderate (kappa coefficient was 0.431), and the intensity of consistency was significantly increased with the assistance of AI (kappa coefficient increased to 0.809), indicating that the diagnostic decisions of the three radiologists were more consistent with the assistance of AI.

## Discussion

In this study, we developed and verified the combined 3 mm radiomic model based on non-enhanced CT, which had better diagnostic efficiency comparing with other models. The three-tiered grading system^4^, which incorporates the heterogeneity and relative proportion of architectural patterns within a tumor based on a histologic pattern, is a strong prognostic classifier of INMA. Previous studies^26^ have pointed out that poorly differentiated grade had a poor prognosis and was predisposed to lymph node metastasis^27^, and our research also confirmed this. In our train cohort and internal test cohort, it was found that grade 3 INMA was more likely to develop lymph node metastasis and vascular invasion than grade 1/2, which may be associated with its poor prognosis.

On the lung CT imaging, the attenuation of nodule could suggest different histopathology of INMA in many researches^12,14,28,29^. We found that the solid nodules were more common in grade 3 INMA (102/116, 87.9%) than grade 1/2 INMA (35/173, 20.2%), and the consolidation size and CTR was larger in grade 3 INMA than grade 1/2 INMA (1.90 cm (1.50–2.40) vs. 0.70 cm (0.00-1.50); 0.90 (0.75-1.00) vs. 0.33 (0.00-0.77)) in the train cohort, and this was kept in line with previous study. Moreover, the clinical model using nodule attenuation, consolidation size and CTR constructed in our study had a good ability to predict poorly differentiated INMA, with AUC values of 0.875 (95%CI 0.811–0.938) and 0.760 (95%CI 0.603–0.916) in the internal and external test cohorts. Although other nodule features^12,15^ such as vacuolation or serum tumor biomarkers were useful in identifying the poorly differentiated grade, nodule attenuation was common feature that affected the degree of differentiation, which was helpful for radiologists in diagnosing pathological subtypes preoperatively.

The strength of our study is the practical application of radiomic features based on non-enhanced CT from intratumoral and peritumoral microenvironment to discern poorly differentiated INMA. Previous research^13^ used the “near-pure” radiomic values (refers to a predominant lung adenocarcinoma sub-type > 70% of tumor volume^30^ and block-by-block image analyses to achieve high sensitivity and moderate specificity for predicting high-grade lung adenocarcinoma, such as solid or micropapillary. The “near-pure” data could reduce the influence of intratumoral heterogeneity and ensure that the extracted histological features were single tissue. According to the new grading system of INMA, there are also some studies^14,15^ using low-dose CT imaging to achieve pathological grade, whose radiomic features were extracted from the tumor itself. Besides, there are studies focusing on the influence of peritumoral microenvironment on the differentiation of pulmonary malignant nodules^31,32^. Niba et al.^17^ document that the peritumoral 5 mm radiomic model is the most effective in differentiating benign from malignant nodules and found interface of the tumor had a “rim” of densely packed tumor-infiltrating lymphocytes and tumor-associated macrophages. Previous studies^33^ have shown that the primary source of heterogeneity in lung adenocarcinoma is the tumor immune microenvironment, which is mainly associated with macrophages and lymphocytes. Based on previous research, our study mainly emphasized on the extraction of the intratumoral and peritumoral 3–5 mm radiomic features, and found that the combined 3 mm radiomic model had the best efficacy for predicting poorly differentiated INMA. However, it is worth noting that the pathological mechanism of better prediction of poorly differentiated INMA by the combined 3 mm radiomic model requires further investigation.

Another important dominance from this study is the adoption of several independent data sets from different hospitals. The external test cohort from two hospitals confirmed the combined 3 mm radiomic model had the higher AUC value, accuracy, specificity and a higher net benefit compared to the other methods, and the constructed model exhibited similar performance in all data sets supporting its robustness. It is interesting to note that the specificity of the model prediction was high and the sensitivity was low in the external test cohort, which could lead to more false negative results. These findings may be related to the limited external data volume, scanner variability, and inconsistencies in reconstruction thicknesses in this study. Therefore, in future prospective multi-center studies, we will develop standardized protocols for data acquisition, standardize data storage and quality control procedures, implement standardized preprocessing pipelines, and establish a robust quality control system to minimize variability risks in the dataset.

In addition to the development of several radiomic models, our study incorporated three radiologists’ evaluations of the internal test dataset with and without the assistance of the combined 3 mm radiomic model. In the trial, it was shown that the radiologists’ final diagnosis improved significantly in the diagnostic efficiency, self-confidence scores and intensity of consistency when the AI-based assistance was consulted. These findings are compatible with the previous study^14^. However, our data demonstrated the benefit of confidence, which used a 5-point Likert scale to quantify, with AI assistance in diagnostic decision. Without the assistance of AI model, radiologists of all ages had low diagnostic efficacy and there was no significant difference between senior radiologist 3 and junior radiologist 2 (*p* = 0.204) in the diagnostic efficacy. This interesting fact also indicates that it’s difficult to identify poorly differentiated INMA only based on the CT image findings of the lesion and the radiologists’ experience before surgery. Therefore, it is urgently needed to exploit advanced technology to help radiologists achieve preoperative precise assessment concerning INMA grade. Furthermore, the model demonstrates robust predictive efficacy for poorly differentiated INMA. For patients stratified as high-risk by the model, radical surgical interventions, including systematic lymph node dissection and lobectomy, should be meticulously considered. Postoperative chemotherapy should also be an integral part of the treatment plan, given its potential to enhance patient outcomes. Conversely, for patients with negative model predictions for poorly differentiated INMA, less invasive sublobar resections like segmentectomy and wedge resection may be viable options. This tailored surgical strategy, guided by the model’s predictions, aims to optimize treatment efficacy while minimizing unnecessary invasiveness.

The study has limitations. First, this study is a retrospective study, and there is a certain selective bias in data inclusion. In light of this, our future work is to conduct a prospective study to control these confounding variables more effectively. Second, the imaging features of manual evaluation and artificially sketched ROI in this study are influenced by individual subjectivity and experience. Finally, this study does not explore the comparison between highly differentiated and moderately differentiated INMA. In the future, we will explore the use of more advanced methods such as deep learning, which will make a more accurate classification of INMA pathological sub-types and reveal the relationship between pathologic grade and genetic status.

In conclusion, this study demonstrates that the combined 3 mm radiomic model based on non-enhanced CT can identify poorly differentiated INMA with excellent performance and this model enable radiologists to achieve more accurate preoperative assessments. In addition, this model has the potential to be a non-invasive preoperative biomarker to precisely predict INMA differentiation degree and the output results can provide guidance for clinicians in selecting appropriate surgical strategies for INMA patients and support personalized treatment planning.

## Electronic supplementary material

Below is the link to the electronic supplementary material.

Supplementary Material 1

## Data Availability

The datasets analyzed and generated during the current study are available from the corresponding author on reasonable request.
