# Supplementary material for: CT radiomics from intratumor and peritumor regions for predicting poorly differentiated invasive nonmucinous pulmonary adenocarcinoma
Source: Sci Rep. 2025 Apr 25;15:14434. doi: 10.1038/s41598-025-99465-z (PMC12032246; doi:10.1038/s41598-025-99465-z)
Supplement: Supplementary file 1 — Supplementary Material 1 [file 41598_2025_99465_MOESM1_ESM.docx]

| Table S1 Summary of characteristics in the external test cohort | | | |
| --- | --- | --- | --- |
| Characteristics | External test cohort (n=38) | | |
|  | Grade 1/2 | Grade 3 | P Value |
|  | (n=26) | (n=12) |  |
| Gender |  |  |  |
| F | 13(50.0) | 7(58.3) | 0.734 |
| M | 13(50.0) | 5(41.7) |  |
| Age(years) | 60.04±7.83 | 63.58±8.23 | 0.590 |
| With smoking history | 9(34.6) | 4(33.3) | 1.000 |
| Lobar position |  |  |  |
| Right upper lobe | 7(26.9) | 6(50.0) | 0.270 |
| Right middle lobe | 1(3.8) | 0(0.0) | 1.000 |
| Right lower lobe | 5(19.3) | 2(16.7) | 1.000 |
| Left upper lobe | 9(34.6) | 3(25.0) | 0.714 |
| Left lower lobe | 4(15.4) | 1(8.3) | 1.000 |
| Nodule attenuation | |  |  |
| Solid nodule | 8(30.8) | 12(100.0) | <0.001* |
| Subsolid nodule | 18(69.2) | 0(0.0) |  |
| Tumor size (cm) | 1.85(1.50-2.32) | 2.85(1.63-3.00) | 0.065 |
| Consolidation size (cm) | 0.90(0.18-1.75) | 2.00(1.20-2.88) | <0.001^*^ |
| CTR(consolidation tumor ratio) | 0.49(0.09-0.85) | 0.92(0.74-0.99) | <0.001^*^ |
| Lobulation | 16(61.5) | 9(75.0) | 0.486 |
| Spiculation | 6(23.1) | 7(58.3) | 0.064 |
| Vacuolation | 21(80.8) | 12(100.0) | 0.158 |
| Pleural indentation | 18(69.2) | 10(83.3) | 0.453 |
| Coursing relationship between bronchus and nodule | | |  |
| Normal | 5(19.2) | 0(0.0) | 0.152 |
| Abnormal | 21(80.8) | 12(100.0) |  |
| Vascular shadow | 21(80.8) | 12(100.0) | 0.282 |

*P<0.05 means that the difference between the two groups was statistically significant. External test group only recorded histopathological classification, lacking detailed pathological information.

| Table S2 Multivariate analysis of logistic in 289 INMA patients in the train cohort. | | | | | | |
| --- | --- | --- | --- | --- | --- | --- |
|  | B | SE | P | OR | 95%CI | |
| nodule attenuation | 3.676 | 0.655 | 0.000* | 39.498 | 10.935 | 142.675 |
| CTR | -2.614 | 1.163 | 0.025* | 0.073 | 0.008 | 0.715 |
| consolidation size | 1.057 | 0.374 | 0.005* | 2.877 | 1.384 | 5.982 |
| constant | -2.234 | 0.353 | 0.000* | 0.107 | - | - |

*P<0.05 means that the difference between the two groups was statistically significant. B: regression coefficient; SE: standard deviation; OR: :odds ratio; 95% CI: confidence interval.

| Table S3 Radiologists’ evaluation in internal test cohort. | | | | | | |
| --- | --- | --- | --- | --- | --- | --- |
|  | Junior radiologist1 | | Junior radiologist2 | | Senior radiologist3 | |
|  | Without AI assistance | With AI assistance | Without AI assistance | With AI assistance | Without AI assistance | With AI assistance |
| Mean confidence | 2.35 | 2.10 | 2.27 | 1.65 | 2.48 | 2.00 |
| Confidence regarding the  diagnosis (n) | 1：18 | 1：32 | 1：18 | 1：57 | 1：20 | 1：40 |
|  | 2：62 | 2：57 | 2：58 | 2：54 | 2：40 | 2：51 |
|  | 3：28 | 3：27 | 3：45 | 3：13 | 3：49 | 3：26 |
|  | 4：15 | 4：7 | 4：3 | 4：0 | 4：14 | 4：7 |
|  | 5：1 | 5：1 | 5：0 | 5：0 | 5：1 | 5：0 |

1 = very certain diagnosis, 2 = certain diagnosis, 3 = likely diagnosis, 4 = uncertain diagnosis, 5 = very uncertain “ambiguous” diagnosis
